# The MDS Mortality Risk Index: The evolution of a method for predicting 6-month mortality in nursing home residents

**DOI:** 10.1186/1756-0500-3-200

**Published:** 2010-07-16

**Authors:** Davina Porock, Debra Parker-Oliver, Gregory F Petroski, Marilyn Rantz

**Affiliations:** 1 School of Nursing, Midwifery and Physiotherapy, The University of Nottingham, Nottingham, UK; 2 Sinclair School of Nursing, University of Missouri-Columbia, Columbia, Missouri, USA; 3 School of Nursing, Edith Cowan University, Perth, Western Australia, Australia; 4 Department of Family and Community Medicine, University of Missouri-Columbia, Missouri USA; 5 Office of Medical Research, University of Missouri-Columbia, Missouri, USA; 6 Sinclair School of Nursing, University of Missouri-Columbia, Missouri, USA

## Abstract

**Background:**

Accurate prognosis is vital to the initiation of advance care planning particularly in a vulnerable, at risk population such as care home residents. The aim of this paper is to report on the revision and simplification of the MDS Mortality Rating Index (MMRI) for use in clinical practice to predict the probability of death in six months for care home residents.

**Methods:**

The design was a secondary analysis of a US Minimum Data Set (MDS) for long term care residents using regression analysis to identify predictors of mortality within six months.

**Results:**

Using twelve easy to collect items, the probability of mortality within six months was accurately predicted within the MDS database. The items are: admission to the care home within three months; lost weight unintentionally in past three months; renal failure; chronic heart failure; poor appetite; male; dehydrated; short of breath; active cancer diagnosis; age; deteriorated cognitive skills in past three months; activities of daily living score.

**Conclusion:**

A lack of recognition of the proximity of death is often blamed for inappropriate admission to hospital at the end of an older person's life. An accurate prognosis for older adults living in a residential or nursing home can facilitate end of life decision making and planning for preferred place of care at the end of life. The original MMRI was derived and validated from a large database of long term care residents in the USA. However, this simplification of the revised index (MMRI-R) may provide a means for facilitating prognostication and end of life discussions for application outside the USA where the MDS is not in use. Prospective testing is needed to further test the accuracy of the MMRI-R and its application in the UK and other non-MDS settings.

## Background

A lack of recognition of the proximity of death is often blamed for inappropriate admission to hospital at the end of an older person's life. An accurate prognosis for older adults living in a residential or nursing home can facilitate end of life decision making and planning for preferred place of care at the end of life. In the UK more than 80% of all deaths occur in the over 65 year old age group and 68% are over 75 years. Furthermore, more than 80% of UK deaths occur in institutions, including more than 20% in the care home setting [1]. Higginson [2] estimates that about three quarters of all deaths are predictable given they occur following a period of chronic illness. Even though chronic illnesses and death are expected in this population, there is still a reluctance to instigate end of life care planning [3,4]. Planning, choice and communication are essential for supporting a good death in which care is individualised and symptoms well managed. Recognising that the patient is at the end of life is the essential first step in accessing and providing excellent palliative care.

General guidance has been provided in the Gold Standards Framework for Community Palliative Care [5,6] prognosis but this has only limited application. However, as Mckillop [7] states, there needs not only to be a general prognosis based on tissue diagnosis and prognostic markers but also an individual prognosis specific to the person sitting before the physician. What is needed now, to complement and operationalise the GSF, is an individualised prognostic "marker" to help initiate these vital conversations about prognosis and EOL care.

The Minimum Data Set (MDS) is federally mandated in the USA for monitoring the quality of long term care in nursing homes certified by Medicare or Medicaid (Health Care Financing Administration, 1995) [8]. The MDS has been used previously to develop predictive models for mortality of residents in relation to specific conditions [9-11] as well as in general [12-15] including our own previous work [16].

In our article in 2005 we reported the development and validation of a predictive model, the MDS Mortality Risk Index (MMRI), of death in 6-months for older adults in nursing homes was published [16]. The model was based on a regression analysis of data from over 43,000 residents taken from Missouri's state MDS data. Two components of the original scoring system made it difficult to implement in practice without the aid of a computer program. Several readers of the article contacted us to enquire if a simplified system could be devised as they wished to trial the scoring system in their own practice and research and in settings where the MDS was not available. Furthermore, it was found that when relying on the usual MDS processing to get the score, too much time had passed and residents had already passed away. Thus a method of scoring that was not reliant on the MDS data collection was needed [17]. The purpose of this paper is to briefly summarise our original work; describe the decision making process associated with simplifying the model; and then report on the performance of the revised MMRI; the MMRI-R.

### Development of the original MDS Mortality Risk Index

The MMRI was developed as a result of a series of studies exploring items relating to mortality using the Minimum Data Set (MDS) for long term care in the USA. Two forms of the MDS are used. The full MDS is a questionnaire with around 400 items which is administered annually or on admission to the long term care facility. A shortened MDS is used quarterly and following any adverse events. The data for the MDS is collected by designated nurses within each facility and are kept centrally in each state and federally. The MDS is primarily used to monitor care quality in long term care facilities and is linked to funding through the Medicare and Medicaid systems in the USA. The MDS includes a broad range of items associated with the health and social wellbeing of residents and although it was not originally designed for research in long term care, the MDS provides a wealth of data and research has developed alongside its use [17,18].

As palliative care researchers our interest began with a single item in the MDS - J5c - that simply stated "The resident has six or fewer months to live" to which a tick in the box indicated the affirmative response and clearly identifying residents known to be at the end of life (EOL). Our first study used admission MDS data to describe residents who were identified as EOL and compare their six month survival with new admissions not so designated [19]. We found only 4% of admissions were designated EOL but that this item was a very good predictor with 50% of these residents dying in the first month of admission and only 17% still being alive 6-months later. Interestingly, 5% of the non-EOL admissions also died in the first month and 15% of them had also died by 6 months. Although J5c was a reliable predictor it was not used frequently enough to make it a useful identifier. Furthermore, our second study revealed that despite the sampling only including residents from facilities that had an active contract with a hospice service only half of the EOL designated residents were receiving input from specialist services [20]. Our conclusion from these studies was that there might be a more reliable way of predicting death using the MDS given the breadth of data available.

On reviewing all items in the MDS we identified 50 that could be related theoretically to the likelihood of death within 6 months. The items were justified by existing empirical research and clinical experience and fell into four categories: demographics (e.g. age and sex); disease (e.g. cancer, chronic heart failure); clinical signs and symptoms (e.g. shortness of breath, poor appetite); and adverse events (e.g. falls, hospitalisation, loss of a spouse). With approval of the University of Missouri institutional review board (IRB) and the appropriate data use agreement, a dataset from the Missouri data was created of residents using the first full MDS (annual or new admission) completed in 1999. The outcome, death of a resident within 6 months of the full MDS assessment, was determined by linking the MDS data to Missouri death certificate data using social security number, sex, and date of birth. This produced a dataset with a full MDS assessment and date of death information for a sample of 43,510 nursing home residents. The death rate in this dataset over the 6 months follow-up was 26%. As described in Porock et al. [16] stepwise logistic regression methods along with a data-splitting strategy was employed to develop a predictive model. The resulting regression model included the following 14 independent variables, listed in priority order for entry into the model: dependency with activities of daily living, shortness of breath, diagnosis of cancer, being an admission assessment, having a poor appetite, being male, general physical deterioration, unintended weight loss over the past 90 days, chronic heart failure, increasing age, renal failure, poor cognitive performance score, diagnosis of Alzheimer's disease or dementia and dehydration. The model also included interaction terms between age and cancer and deterioration and admission, giving a final model with 16 terms. A point-value for each risk factor was derived by transforming the associated logistic regression coefficients to integer values, which are then summed to give the MMRI score. Depending on the selected "cut-point" the summated scale scores can provide a highly sensitive or highly specific instrument.

In developing the original MMRI, the goal was to provide simple yet accurate instrument that could predict short term mortality. In practice the calculation of a hierarchical Activities of Daily Living (ADL) score [21] and the Cognitive Performance Scale (CPS) [22] were too complex to be easily implemented by hand. As the authors and others attempted to use the MMRI we found using the algorithms too unwieldy and time consuming [23]; the computations were confusing and the number of variables excessive.

The modification of the MMRI was undertaken with two research questions in mind. First, can a simpler ADL and Cognitive performance measure produce an equally strong model? Secondly, can we eliminate some of the variables while maintaining predictive validity?

## Methods

### Modifying the MMRI

With the opportunity to review the MMRI we carefully considered the theoretical and statistical implications of each item as well as the practical issues of dealing with the ADL and CPS scores. The original MMRI included the item "deterioration" which was based on MDS item Q2 (Overall Change in Care Needs). On review, the definition of this item seemed vague with a high risk of poor reliability between raters. We decided to try the model without this variable

#### Modifying the ADL score

The MDS includes a number of ordinal items the reflect the degree of assistance required over the previous seven days in performing typical activities of daily living (ADL) such as bed transfers, eating, dressing and locomotion. Items are scored on a 5 point scale: 0 = independent to 4 = total dependence. The MDS provides clear definitions of the ordinal scaling (see Additional File 1). Morris et al. [21] proposed a variety of ways of aggregating the items into ADL scales. Based on face validity the MMRI adopted the hierarchical ADL scale [21] but in practice this proved too complex for routine use. Thus we considered replacing it with a simple summated scale and considered as alternatives the long-form, short-form, early-, middle-, and late-loss ADL scales proposed by Morris et al. [21]. We decided to use the short-form because it was parsimonious and valid.

#### Modifying the CPS score

Calculation of the CPS [22] was another aspect of the original MMRI that proved too cumbersome for routine use without software. Five individual MDS items were considered as replacements for the CPS: Indicators of short- and long-term recall (MDS items b2a & b2b), a single ordinal item on impairment in daily decision making (b4), and an item reflecting stability, improvement or deterioration in cognitive skills over the previous 90 days (b6). The cognitive-change item was recoded to an indicator of cognitive deterioration versus no-change or improvement. Although the Alzheimer's/Dementia diagnosis variable had been predictive in the original analysis we decided not to include it as the important factor in predicting mortality is the change in cognitive function rather than the formal diagnosis.

Following a procedure essentially identical to those used to develop the original risk index and using the same raw data set, the total data was split randomly 75%/25% into development and validation data sets. Twenty random subsets each consisting of approximately 10,800 subjects were drawn from the development data. Logistic regression with stepwise selection was performed on each subset and the frequency and entry step of each candidate replacement variable was recorded. Selection on multiple subsets of the data help to avoid the biases and 'fluke' results associated with variable selection methods [24]. Following selection of the replacements for the hierarchal ADL scale and CPS, two way interactions involving the new predictors were examined.

## Results

### Validating the new MMRI

The Short-Form ADL Scale [21] was the first variable to enter on 19 of the 20 random subsets and so this four item scale was adopted as the replacement for the hierarchical ADL scale. Of the candidate replacements for the CPS the MDS item for change in cognitive status (b6) was the most frequently selected cognitive variable entering on 12 of 20 subsets. There was not a statistically significant difference (p > 0.05) in the probability of death for those with stable versus improving cognitive status and so the item was recoded to an indicator of cognitive deterioration. The only interaction effect added to the model was between cognitive deterioration and the new ADL scale. In the revised model age categories were revised to a uniform set of five year increments.

Table 1 gives the regression model used to derive the MMRI-R points system. Area under the ROC curve is a commonly used measure of a model's ability to discriminate between observed outcomes. For the revised regression model the ROC area was 0.76 and Hosmer-Lemeshow goodness-of-fit test [25] indicated adequate fit with p = 0.16. As a final step in evaluating the regression model we fit a logistic regression model with six month mortality as the outcome and the MMRI-R score as the only independent variable.

**Table 1:** Revised Logistic Regression Model - Development Data

			95% Confidence Limits	
Variable	Regression Coefficient	Odds Ratio	Lower	Upper
Intercept	-5.523	0.00	0.00	0.01
Sex	0.550	1.73	1.64	1.83
Admission	0.760	2.14	2.02	2.26
Shortness of Breath	0.820	2.27	2.11	2.45
Appeite	0.411	1.51	1.42	1.60
Weight Loss	0.459	1.58	1.48	1.69
Congestive Heart Failure	0.362	1.44	1.36	1.52
Renal failure	0.645	1.91	1.68	2.16
Dehydrated	0.402	1.49	1.33	1.68
Cancer	5.138			
Age	0.026			
Cancer*Age	-0.051			
ADL	0.104			
Cognitive Deterioration	-0.171			
ADL * Cognitive Deterioration	0.045			

Note: Odds ratios not calculated for variables included in interaction terms.

**Figure 1:**
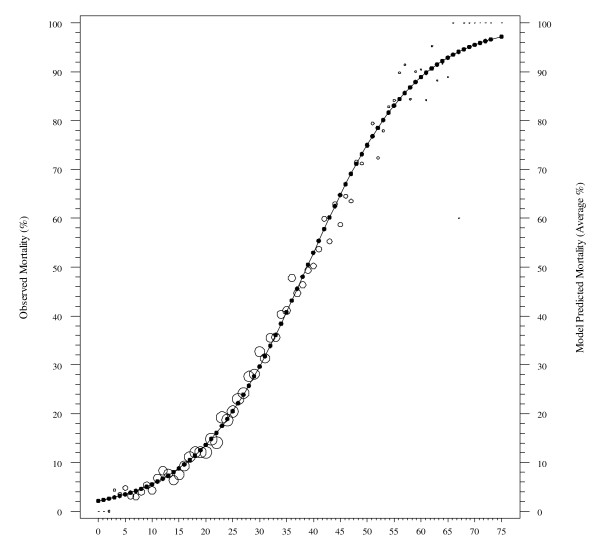
**Observed and Predicted Six Month Mortality by MMRI-R Score - Validation Data**. Open circles denote observed mortality. Dots denote model-predicted average mortality and MMR-R value.

To form the MMRI-R scoring system, weights were assigned to each variable by rescaling the estimated regression coefficients to integer values. Tables of tabulated values for the interaction effects suggested cut-points for these terms. The Additional File 1 gives the revised point system. The theoretical range of MMRI-R is 0 to 85 with larger values indicating greater short-term mortality risk. For our sample the mean score was 24 with a standard deviation of 10 points and a range from 0 to 75. The Spearman correlation between risk scores on the original and revised MMRI was 0.95 (p < 0.0001). Figure 1 displays the observed percent of residents who died and the model-predicted mortality at each value of MMRI-R score. Overall agreement is quite good with deviations mostly at the extreme end of the score range where death within six months is almost certain. This indicates that the complexity of the original regression model is captured well in the MMRI-R score. Area under the ROC curve for the single variable model is identical to the original model.

Table 2 provides the frequency distribution of MMRI-R values and six month mortality for five point increments of the MMRI-R score distribution. Figure 1 is a plot of the conditional probability of death given each MMRI-R score or one greater. Thus for example, the estimated probability of dying within six months given an MMRI-R score of 34 or greater is .40 and and with a score of 50 or greater the probability is approximately 0.80. Six month mortality in the total sample is 23.3% and so this is the value associated with an MMRI-R ≥ 0. Table 3 provides estimates of sensitivity, specificity, predictive value positive (PVP), and predictive value negative (PVN) rates as well as a calculation of Predictive Summary Index (PSI) [26] when a prediction of death within six months is made using the low boundary of the categories in the table as decision points. For example, if an MMRI-R score of 41 *or greater *is used as the criterion value, then one would expect this prediction to be almost 99% specific but not very sensitive (11.3%). The choice of a cut-point will depend on what types of errors are most important for the circumstance. In combination Additional File 1 and Table 3 should be useful for choosing a decision rule for a particular application. Sensitivity and specificity are informative measures of a predictive or diagnostic test's performance however they do not directly address the questions of greatest interest to the clinician, namely given a test result, or MMRI-R score in this study, what is the probability of survival. PVP and PVN rates are more useful from the clinicians perspective in determining the correctness of a decision than the sensitivity and.

**Table 2:** Frequency Distribution of MMRI-R Scores and Mortality by 5-point Intervals - Development data for 6 month mortality

MMRI-R Risk Group	Sample Size	Percent of Sample	Number of Deaths	Percent Died
Overall	43311	100	10025	23
0-5	627	1.4	25	4
6-10	2986	6.9	120	4
11-15	5271	12.2	387	7
16-20	7706	17.8	881	11
21-25	8812	20.3	1530	17
26-30	7173	16.6	1925	27
31-35	4703	10.9	1710	36
36-40	2903	6.7	1377	47
41-45	1628	3.8	939	58
46-50	832	1.9	570	69
51-55	382	0.9	302	79
56-60	177	0.4	158	89
61-65	78	0.2	70	90
66-70	28	0.1	26	93
71-75*	5	0.0	5	100

* In our validation dataset the highest score is 75 however theoretically the score could be up to 85 which occurred in the development dataset. Given that all residents scoring over 70 died in the subsequent 6-months we would anticipate that scores 76 to 85 would also indicate an extremely high likelihood of death.

**Table 3:** Sensitivity, Specificity, and Predictive Value Positive (PVP) and Predictive Value Negative (PVN) Rates, and Predictive Summary Index (PSI) for Different MMRI-R "Cut-Points" - Validation Data

MMRI-R Cut-Point	Sensitivity	Specificity	PVP	PVN	PSI
0	100.00		23.15		
6	99.75	1.81	23.43	96.01	0.19
11	98.55	10.42	24.89	95.99	0.21
16	94.69	25.09	27.57	94.01	0.22
21	85.91	45.60	32.23	91.48	0.24
26	70.64	67.47	39.54	88.41	0.28
31	51.44	83.24	48.03	85.06	0.33
36	34.38	92.23	57.14	82.35	0.39
41	20.65	96.82	66.13	80.20	0.46
46	11.28	98.89	75.30	78.73	0.56
51	5.60	99.67	83.73	77.81	0.62
56	2.58	99.91	89.93	77.30	0.67
61	1.01	99.97	90.99	77.03	0.68
66	0.31	99.99	93.94	76.91	0.71
71	0.05	100.00	100.00	76.86	0.77

Table notes:

• Sensitivity is the probability of observing an MMRI-R score greater than or equal to the cut-point given mortality within six months.

• Specificity is the probability of observing an MMRI-R score less than the cut-point given survival beyond six months.

• Predictive value positive (PVP) and is the probability of dying within six months given an MMRI-R score is greater than or equal to the cut point.

• Predictive value negative (PVN) and is the probability of surviving beyond six months given an MMRI-R score less than the cut-point.

• The appropriateness of PVP and PVN depends on having a good estimate of population prevalence, in this case the prevalence of six month mortality.

• For ease of interpretation all probabilities were converted to percentages.

• Predictive Summary Index (PSI), calculated as PVP + PVN -100, is a overall measure of the gain in certainty associated with using the MMRI-R to make decisions. The practical range of PSI values is 0 to 1 with 0 reflecting a useless test and PSI = 1 for a test yielding perfect predictions.

## Discussion

### Using the MMRI-R

The MMRI-R is currently being tested as part of a 5 year NIHR programme grant (National Institute for Health Research; PI Prof J Gladman). A potential limitation of the model may be that we do not know the validity of the definitions of items in the MDS for practice. The current study will help to determine the answer to that question. However, the new predictive model demonstrates a similar accuracy to the original MMRI and the modifications have been made with the same balance of statistical rigor and clinical application.

The MMRI-R (Additional file 1) is designed to fit on two sides of a single sheet of paper where the score can be easily calculated by summing the relevant weighted scores along with the calculated scores for the interaction terms: "age and cancer"; and "ADL and cognitive decline". At the bottom of the first page is a table indicating the percentage of deaths in the original dataset for individual scores divided into 5-point bands. From this, a health professional could easily judge the likely prognosis of the older person they are assessing.

For example, John, 72 years old, has been living in the nursing home for about 6 months following the death of his wife a year ago. John has CHF and some further cognitive deterioration over the past three months and is scoring 7 (mobility = 2; eating = 1; toilet use = 2; personal hygiene = 2) on the ADL score but has a good appetite would give a MMRI-R score of 19. From the data set 11% of comparable older adults scoring 19 died in the following 6 months. John's daughter wants to know if he should undergo knee replacement surgery.

Another example is Marion, 75 years old with colon cancer. She has become short of breath, is not so interested in food lately and she has lost some weight. She has lived in the nursing home since her husband died two months ago because without her husband to care for her, her arthritis makes it increasingly difficult to manage everyday activities. Marion's ADL score was 12 (mobility 3, eating 3, toilet use 3; personal hygiene 3) making her total MMRI-R score 50. From the Missouri dataset, 69% of comparable older adults died in the following 6 months. Marion's pain and shortness of breath is increasing and she is asking what this means. The MMRI-R scores would help health professionals initiate conversations with John and Marion and provide meaningful information for decision making about treatment choice and preferences.

Of course there remain aspects of the assessed items that are potentially reversible e.g. dehydration and others potentially treatable for example shortness of breath may be reduced with diuretics. These treatment decisions would be part of the end of life care discussion. The MMRI-R thus acts as a prompt for review of current treatment as well as a prompt for EOL care planning. The cut off score for suggesting that palliative goals of care be discussed has not been determined and is for future research to inform.

## Conclusion

The MMRI-R is not designed nor has it been tested to be any more than an aid to clinical judgement. The model does not calculate when or of what an older person in a long term care facility will die. It does not provide a definitive prediction of whether someone will fall or acquire a chest infection that ultimately turns out to be the terminal event. What the MMRI-R score may provide is an indication of risk of death when compared with older adults in similar condition, the purpose of which is to trigger conversations with residents and families and assist in providing some context for end of life care planning.

Although using a prognostic score for death in older residents may seem unusual, even distasteful, a salutary lesson can be learned from the management of pain. A commonly accepted practice across all settings of healthcare is the use of a simple visual analogue scale for measuring pain; where 0 or 1 is no pain and 10 is the worst pain imaginable. The simplicity of the tool and the consolidation of assessment can quickly spur practitioners to respond confidently and appropriately. The benefits of being able to have a reasonably accurate prediction of prognosis that could similarly spur practitioners into having EOL care planning conversations at the appropriate time include: promoting individual choice in type and place of care; to reductions in unnecessary admissions to hospital; and initiation of futile treatments. We do not know what the impact of giving professionals, residents or families a score like this would have on decision making and planning for end of life care. That will need to be the subject of further research with which we hope this simplified method will assist.

## Abbreviations

MMRI: MDS Mortality Risk Index; MMRI-R: MMRI-Revised; MDS: Minimum Data Set.

## Competing interests

The authors declare that they have no competing interests.

## Authors' contributions

The following contributions were made by each author. DP contributed to the conception of the research, interpretation of findings and drafting of the paper. DPO also contributed to the conception of the research and the interpretations of findings. GP was responsible for data management and analysis, and production of findings. MR provided access to the MDS database and consultation on research. All authors were involved in editing of the paper and have read and approved the final manuscript.

## Authors' information

Davina Porock RN PhD

Professor of Nursing Practice, The University of Nottingham

Adjunct Professor at University of Missouri and Edith Cowan University, Perth, WA

Davina Porock RN PhD

The University of Nottingham

B46, South Block, Queen's Medical Centre

Nottingham, NG7 2HA, UK

+44 (0)115 8230813 (tel)

+44 (0)115 8231208 (fax)

http://davina.porock@nottingham.ac.uk

Debra Parker-Oliver MSW PhD

Associate Professor, Department of Family and Community Medicine

University of Missouri-Columbia, USA

Gregory F. Petroski PhD

Biostatistician, University of Missouri-Columbia, USA

Marilyn Rantz RN PhD FAAN

Professor of Nursing, Sinclair School of Nursing

University of Missouri-Columbia, USA

## Supplementary Material

Additional file 1 **the MMRI-R**. The file contains the MMRI-R scoring sheet. Click here for file
