# Supplementary material for: The MDS Mortality Risk Index: The evolution of a method for predicting 6-month mortality in nursing home residents
Source: BMC Res Notes. 2010 Jul 16;3:200. doi: 10.1186/1756-0500-3-200 (PMC2913927; doi:10.1186/1756-0500-3-200)
Supplement: Additional file 1 — the MMRI-R. The file contains the MMRI-R scoring sheet. [file 1756-0500-3-200-S1.DOC]

###

**The MDS Mortality Risk Index – Revised (MMRI-R)**

Weighted

points

Admission to nursing home in the past three months Yes 🞏 No 🞏^*^ (8)

Lost weight unintentionally in the last three months Yes 🞏 No 🞏 (5)

Renal failure Yes 🞏 No 🞏 (6)

Chronic heart failure Yes 🞏 No 🞏 (4)

Poor appetite Yes 🞏 No 🞏 (4)

Male Yes 🞏 No 🞏 (5)

Dehydrated Yes 🞏 No 🞏 (4)

Short of breath Yes 🞏 No 🞏 (8)

Cancer (if yes – see Age and Cancer worksheet; if no continue) Yes 🞏 No 🞏**

Age of patient/resident at last birthday Age score **without** cancer (2-9)

Age score **with** cancer (13-20)

Deteriorated cognitive skills or status in the past three months Yes 🞏 No 🞏***

Activities of Daily Living score ADL score **without** cognitive decline (0-16)

(see ADL and cognitive decline worksheet) ADL score **with** cognitive decline (-2-21)

**TOTAL MMRI-R SCORE (0-85)**

* If “yes” then score 8 – if “no” then score zero. This system applies to the first eight items using the indicated weighted scores.

** Age must be scored with or without a diagnosis of active cancer – use worksheet to calculate score

*** ADLs must be scored with or without cognitive deterioration in the past three months – use worksheet to calculate score

**Percentage of deaths in 5-point bands**

| **Points** | **% died** |
| --- | --- |
| 0-5 | 4 |
| 6-10 | 4 |
| 11-15 | 7 |
| 16-20 | 11 |
| 21-25 | 17 |
| 26-30 | 27 |
| 31-35 | 36 |
| 36-40 | 47 |
| 41-45 | 58 |
| 46-50 | 69 |
| 51-55 | 79 |
| 56-60 | 89 |
| 61-65 | 90 |
| 66-70 | 93 |
| 71-75 | 100 |

**WORKSHEETS**

Age and Cancer worksheet

| **Age** | **Score without cancer** | **Score with Cancer** |
| --- | --- | --- |
| <=69 | 2 | 20 |
| 70-74 | 3 | 19 |
| 75-79 | 4 | 18 |
| 80-84 | 5 | 17 |
| 85-89 | 6 | 15 |
| 90-94 | 7 | 14 |
| 95-99 | 8 | 14 |
| 100+ | 9 | 13 |

**ADL and Cognition worksheet**

Circle the level of independence the patient/resident has in these ADLs using the following five point scale in the past seven days.

| Mobility/locomotion   1. Independent 2. Supervised 3. Limited assistance 4. Extensive assistance 5. Total Dependence | Eating   1. Independent 2. Supervised 3. Limited assistance 4. Extensive assistance 5. Total Dependence | Toilet use   1. Independent 2. Supervised 3. Limited assistance 4. Extensive assistance 5. Total Dependence | Personal hygiene   1. Independent 2. Supervised 3. Limited assistance 4. Extensive assistance 5. Total Dependence |
| --- | --- | --- | --- |

INDEPENDENT = No help or oversight – OR – help or oversight provided only 1 or 2 times during the last 7 days.

SUPERVISED = Oversight, supervision or cueing provided 3 or more times during the last 7 days – OR – Supervision 3 or more times plus limited assistance provided only 1 or 2 times during the last 7 days.

LIMITED

ASSISTANCE = Patient/resident highly involved in activity: received physical help in guided manoeuvring of limbs or other non-weight bearing assistance 3 or more times – OR – More help provided only 1 or 2 times in the last 7 days.

EXTENSIVE

ASSISTANCE = While patient/resident performed part of activity over last 7 day period help of the following types provided 3 or more times:

- weight bearing support
- full staff performance during part (but not all) of the past 7 days

TOTAL

DEPENDENCE = Full staff performance during past 7 days

**ADL score *WITHOUT* cognitive deterioration is the sum of points assessed for Mobility + Eating + Toilet Use + Hygiene**

**ADL score *WITH* cognitive deterioration (sum the ADLs as above and then use the table to assign the score)**:

| **ADL Points Score** | **ADL Point Score with Cognitive Decline** |
| --- | --- |
| 0 | ADL score – 2 |
| 1,2 | ADL score – 1 |
| 3,4 | ADL score |
| 5,6,7 | ADL score + 1 |
| 8,9 | ADL score + 2 |
| 10,11 | ADL score + 3 |
| 12,13 | ADL score + 4 |
| 14,15,16 | ADL score + 5 |
